# Dendritic-cell-targeting adeno-associated virus as potent cancer vaccine carriers

**DOI:** 10.3389/fimmu.2026.1932907

**Published:** 2026-09-30

**Authors:** Xin Wei, Xiang Li, Lixia Qi, Wenxuan Qiu, Yuxin Zhang, Liping Wu, Shutang Wei, Bofeng Luo, Menghan Shi, Chenhao Liu, Qi Yang, Wenyi Yang, Dengke Bao

**Affiliations:** 1 The Department of Gastroenterology, The First Affiliated Hospital of Henan University, Kaifeng, Henan, China; 2 Joint National Laboratory for Antibody Drug Engineering, School of Basic Medical Sciences, Henan University, Kaifeng, Henan, China; 3 Laboratory of Cancer Biomarkers and Liquid Biopsy, School of Pharmacy, Henan University, Kaifeng, Henan, China

**Keywords:** adeno-associated virus, antigen gene delivery, cancer vaccines, dendritic cells, tumor immunotherapy

## Abstract

**Background:**

The clinical efficacy of therapeutic cancer vaccines is critically limited by the inefficient *in vivo* delivery of tumor antigens to dendritic cells (DCs), the key regulators of T-cell immunity.

**Methods:**

To address this issue, we developed a dendritic-cell-targeting adeno-associated virus (AAV) vector through rational capsid design. This was achieved by site-specifically inserting the high-affinity Clec9a-targeting peptide CBP-12, optimizing its flanking sequences, and introducing T491V/S662V gain-of-function mutations to synergistically enhance DC transduction.

**Results:**

The resulting vector, AAV2-mut-Peptide2, showed significantly improved tropism for and gene delivery efficiency to cross-presenting cDC1s *in vivo*. In murine models of hepatocellular carcinoma, vaccination with this platform, encoding either a model antigen (OVA) or the endogenous tumor-associated antigen GPC3, elicited a potent antigen-specific CD8+ T cell response. This response was characterized by a substantial increase in tumor-infiltrating lymphocytes, enhanced production of key effector cytokines, and consequently, potent suppression of tumor growth.

**Conclusion:**

Our study establishes this engineered AAV as a versatile and potent vaccine platform that effectively targets DCs, offering a promising approach for inducing robust and adaptable anti-tumor immunity.

## 1 Introduction

Cancer poses a significant global health challenge, with 20 million new cases and 9.7 million deaths in 2022 (1). Despite progress in surgery, chemotherapy and radiotherapy, many cancers are still incurable particularly in advanced stages where options are limited. Moreover, the lack of specificity in targeting tumor cells and side effects from chemotherapy and radiotherapy have severely compromised patient health. Recently, immunotherapies such as immune checkpoint inhibitors (ICIs), adoptive cell transfer therapy (ACT) have emerged as promising strategies to harness the immune system against malignant tumors (2–4). Although these approaches have shown excellent efficacy in some patients, their success remains limited, with only a fraction of patients achieving sustained remission. In contrast, therapeutic cancer vaccines offer distinct advantages: they stimulate antigen-specific cellular and humoral immune responses *in vivo*, effectively inhibiting tumor growth and eliminating tumor cells with fewer side effects (5, 6). Cancer vaccines can function as standalone treatments or complement existing immunotherapies, potentially transforming the current cancer treatment paradigm.

Despite numerous studies and advances in tumor vaccine research, clinical trials of therapeutic cancer vaccines have yet to produce promising results (7, 8). A primary obstacle is their inadequate delivery and stimulation of antigen-presenting cells (APCs). The APCs population includes dendritic cells (DCs), macrophages, and B cells, but DCs are considered the most effective APCs in initiating cytotoxic and memory T-cell responses through cross-presentation (9–11). Currently, the DC-based vaccine Provenge has been approved by the FDA for the immunotherapy of prostate cancer. However, its requires isolating APCs from patients and activating them ex vivo. This process is cumbersome, technically challenging, and expensive, hindering its broader application. Additionally, some cancer mRNA vaccines lack targeting specificity toward APCs, leading to substantial tumor antigen expression in non-professional APCs. This may result in antigen-nonspecific presentation, triggering CTL-mediated off-target toxicity and increasing treatment-related side effects (12–14). Therefore, achieving efficient and specific *in vivo* targeting of DCs to enhance the specificity and safety of immune responses has become one of the key focus in current cancer vaccine design.

Among various DC subsets, conventional type 1 DCs (cDC1s) stands out due to its exceptional ability for cross-presentation, particularly in activating antitumor immune responses (11, 15). Notably, C-type lectin domain family 9 member A (Clec9a), a specific surface molecule, is almost exclusively expressed on the cDC1s. This receptor significantly enhances the efficiency of cross-presentation by recognizing filamentous actin exposed by dead or damaged cells, thus crucially initiating and amplifying CTL responses (15–18). Gou et al. developed a 12-mer peptide (CBP-12) with high affinity for Clec9a, which can serve as a vaccine carrier targeting cDC1 to effectively enhance antigen delivery (19). However, even when conjugated with tumor antigen peptides, CBP-12 encounters two major challenges *in vivo*: first, the conjugate is prone to degradation, resulting in insufficient stability; second, this strategy primarily achieves targeted antigen delivery but provides limited stimulation for the activation and maturation of cDC1 itself, thereby failing to fully elicit its antigen-presenting function and co-stimulatory signals, which restricts the generation of potent CTL responses. Given the limitations of peptide vaccines, especially those based on targeted peptides (e.g., CBP-12), in terms of *in vivo* stability and DC activation, exploring and developing novel vaccine platforms is of paramount importance.

Viral vector vaccine platforms offer distinct advantages by delivering genes encoding target antigens, utilizing the host cell’s machinery for sustained endogenous antigen expression. This approach not only overcomes the challenge of peptide degradation but also mimics the natural antigen presentation pathway following viral infection, particularly facilitating cross-presentation via the MHC class I pathway (20–22). Among viral vectors, the Adeno-Associated Virus (AAV) vector is particularly promising for clinical use due to its non-pathogenicity, low immunogenicity, and ability to ensure long-term stable transgene expression (23–26). Several AAV vector-based drugs have already been approved for treating various genetic and rare diseases, preliminarily validating their safety and efficacy (27).

However, Wild-type AAV vectors present notable limitations. They show a strong tropism for highly perfused tissues such as the liver, which may lead to off-target delivery to non-target cells or tissues (28). This can result in off-target toxicity and aberrant antigen expression that deviates from the intended immune targets, compromising therapeutic safety and diluting effective immune responses. Additionally, AAV vectors have low transduction efficiency in immune cells, especially professional APCs like DCs, which hinders adequate antigen loading and presentation (26, 29). This significantly limits their utility in therapeutic cancer vaccines.

Here, we engineered and optimized the AAV capsid to develop an efficient gene delivery vector targeting DCs and evaluated its potential for immunotherapy in hepatocellular carcinoma. For AAV capsid modification, we primarily utilized the Clec9a-affinity peptide CBP-12, incorporating it into the AAV capsid while optimizing the amino acid sequences flanking the insertion site. This modification not only theoretically disrupts the heparan sulfate proteoglycan (HSPG) binding region of the viral particle, but also facilitated enhanced exposure of CBP-12 on the viral surface, thereby improving DC-targeting by the AAV vector. Based on previous studies, we substituted threonine at position 491 and serine at position 662 with valine, further enhancing DC transduction efficiency and CD8^+^ T cell activation (30). *In vitro* and *in vivo* tests showed that the recombinant AAV (rAAV) exhibited higher transduction efficiency in DCs compared to wild-type AAV and effectively delivered vector antigens. Subsequently, by establishing an OVA-expressing Hepa1–6 cell line and a subcutaneous Hepa1–6 tumor model, we confirmed that the modified rAAV vector significantly improved antigen presentation efficiency, increased immune cell infiltration in tumor tissues, and effectively suppressed tumor progression. In summary, we constructed a highly efficient DC-targeting antigen delivery system based on AAV, which simultaneously overcomes the inherent limitations of both non-targeted vaccines and conventional AAV vectors, providing a novel strategy for cancer vaccine development.

## 2 Materials and methods

### 2.1 Vector construction

For the modification of AAV coat proteins, PCR is employed to insert a polypeptide at position R588 of the VP1 and to mutate specific amino acid residues, and the specific modification forms of each different AAV variant are as follows. AAV2-Peptide1: inserts CBP12 at position R588 of AAV2 VP1. AAV2-Peptide2: inserts CBP12 at position R588 with upstream and downstream sequences changed to QGRG and QA, respectively. AAV2-mut: mutates residues T491 and S662 of AAV2 VP1 to valine. AAV2-mut-Peptide1: mutates residues T491 and S662 to valine and inserts CBP12 at position R588. AAV2-mut-Peptide2: mutates residues T491 and S662 to valine, inserts CBP12 at position R588, and changes flanking sequences to QGRG and QA, respectively. Specifically, the AAV packaging plasmid pR2C2 was subjected to double enzymatic cleavage (MluI and BamHI), followed by the design of different primers to obtain DNA fragments of the desired modified form by PCR, and then ligated the desired fragments using seamless cloning. Amino acid sequences of the various modified AAV VP1 were shown in **Supplementary Table S1**. It was confirmed that all the modifications were correct by DNA sequencing.

### 2.2 Cell lines

HEK 293T cell line was purchased from Shanghai Saibikang Biotechnology Co., Ltd. The DC 2.4 cell line was a gift from Prof. Z.Y Yan. And mouse hepatoma Hepa 1–6 cell line was purchased from Wuhan Servicebio Technology Co., Ltd. Cells were cultured in high-glucose DMEM medium supplemented with 10% FBS and 1% penicillin-streptomycin, and maintained in a 37 °C, 5% CO_2_ incubator. HEK 293T cells were co-transfected with pMD2.G, psPAX2, and pAAV-OVA plasmids using Lipofectamine 2000 transfection reagent. For Hepa 1-6-OVA cells The viral supernatant was collected and used to infect Hepa 1–6 cells. After 48 hours, the cells were selected with puromycin for two weeks. OVA expression was verified by Western blot and RT-qPCR.

### 2.3 Production of recombination AAV vectors

AAV production by triple transfection involves the transfection of three plasmids into cells to produce AAV vectors. The first plasmid contains the AAV genome (pAAV), the second encodes for the AAV Rep and Cap proteins (pR2C2), and the third plasmid provides helper functions needed for the production of viral particles (pHelper). For production of rAAVs, HEK 293T cells were seeded into several 100 mm diameter dishes, and transfected 24 h later using Polyethylenimine (PEI, linear, MW 25,000, beyotime) transfection reagent and following the manufacturer’s instructions for this format. The transfection mixture contained transfer vector pAAV, packaging plasmid pR2C2 and helper plasmid pHelper were mixed in a ratio of 3:5:6. Cells were scraped off, pelleted and lysed 72 h after transfection. All rAAV types were purified by ultracentrifugation through an iodixanol density gradient followed by buffer exchange using Poloxamer 188/PBS buffer via a 50 kDa Amicon Ultra-4 centrifugal filter unit (Merck Millipore, UFC805024). The genomic titers of the purified AAV vectors were determined by quantitative real-time PCR using Power SYBR Green Master Mix (Roche) with the primers F-5’-CTGTTGGGCACTGACAATTC-3’, R-5’-GAAGGGACGTAGCAGAAGGA-3’ targeting the WPRE sequence. Expression plasmid vectors were used as standards. The purified AAV vectors were stored in aliquots at −80 °C for later use.

### 2.4 Negative stain transmission electron microscopy

AAV particles were immobilized on nickel grids and stained with 2% phosphotungstic acid (PTA) for 1 min. Samples were visualized using electron microscope. Brightness and contrast of images were adjusted using ImageJ retaining relative signal strength.

### 2.5 Quantitative real-time PCR

Total RNA was extracted from cells or tissues using TRIzol LS reagent according to the manufacturer’s instructions. Tissue samples were mechanically homogenized. RNA concentration and purity were determined using a NanoDrop 2000 spectrophotometer. cDNA was synthesized from total RNA using the 5×Evo M-MLV RT Master Mix (Nanjing Vazyme Biotechnology Co., Ltd.). qPCR was performed in a 10 μL reaction volume. The relative mRNA expression levels of target genes (OVA, EGFP, IFN-γ, IL-6, TNF-α) were normalized to GAPDH and calculated using the 2^^^(-^ΔΔ^Ct) method.

### 2.6 Flow cytometry

For cell lines, cells were harvested, washed with PBS, and blocked with 3% BSA. For tissues, lymph nodes and tumor tissues were minced, digested with collagenase IV and DNase I at 37 °C for 1 hour, and passed through a 70 μm cell strainer to prepare single-cell suspensions. Red blood cells were lysed. Cell suspensions were blocked and then stained with fluorochrome-conjugated antibodies (e.g., CD11c-PE, CD3-FITC, CD8-APC, IFN-γ-PE-Cy7) for 30 minutes at 4 °C in the dark. After washing, cells were analyzed on a Beckman Coulter flow cytometer. Data were analyzed using FlowJo software to determine the percentages of specific cell populations. The detailed gating strategy is provided in **Supplementary Figure S5**.

### 2.7 Western blot

Cells were lysed in RIPA buffer on ice. The lysates were centrifuged, and the protein concentration of the supernatant was determined using a BCA assay. Protein samples were denatured in 5× loading buffer at 100 °C. Proteins were separated by SDS-PAGE and transferred onto a polyvinylidene fluoride (PVDF) membrane. The membrane was blocked with 5% skim milk and then incubated with primary antibodies (against OVA, VP1/VP2/VP3, GAPDH) overnight at 4 °C. After washing, the membrane was incubated with an HRP-conjugated secondary antibody. Protein bands were visualized using an enhanced chemiluminescence (ECL) substrate and imaged.

### 2.8 Immunofluorescence staining

Mouse lymph node sections were baked in a temperature-controlled oven to improve adhesion. After permeabilization at room temperature and washing, the tissue sections were outlined with a hydrophobic barrier using an immunohistochemistry pen. The enclosed area was blocked with 3% bovine serum albumin (BSA) in a humid chamber at room temperature for 1 h to reduce nonspecific binding. Sections were then incubated with diluted primary antibody at 4 °C overnight. After returning to room temperature, sections were incubated with fluorophore-conjugated secondary antibodies. Following washes, nuclei were counterstained with DAPI in the dark. Finally, sections were mounted with anti-fade medium, coverslipped, and imaged by fluorescence microscopy.

### 2.9 Animal study

Five-week-old male C57BL/6J mice were purchased from Henan SCBS Biotechnology Co., Ltd. (License No. SCXK (Yu) 2020-0005) and housed under SPF conditions. All animal experiments were approved by the Institutional Animal Care and Use Committee. For surgical procedures, mice were anesthetized by intraperitoneal injection of tribromoethanol (Avertin) at 250 mg/kg body weight. The anesthetic solution was prepared as a 2.5% working stock and stored at 4 °C in the dark prior to use. Animals were fasted for 6–8 h with free access to water before anesthesia. Anesthesia depth was confirmed by the absence of toe pinch and corneal reflexes. During recovery, mice were kept warm and closely monitored until fully conscious. At experimental endpoints, mice were euthanized by intraperitoneal injection of tribromoethanol at 500 mg/kg, followed by cervical dislocation to ensure death. Cessation of respiration, absence of corneal reflexes, and cardiac arrest were confirmed and observed for at least 5 min before tissue collection. Euthanasia was performed in accordance with the AVMA Guidelines for the Euthanasia of Animals.

Hepa 1–6 or Hepa 1-6-OVA cells in the logarithmic growth phase were harvested and subcutaneously injected into the scapular region of each mouse. Treatments were initiated via tail vein injection nine days post-inoculation. Mice received rAAV at a dose of 1×10^11^ genome copies per kg, administered once every three days. Tumor dimensions (length and width) were measured weekly, and tumor volume was calculated. After 21 days of treatment, mice were euthanized. Tumors, lymph nodes, and major organs (heart, liver, spleen, lung, kidney) were collected. Tissues were either fixed in 4% paraformaldehyde for histological analysis or snap-frozen in liquid nitrogen for molecular studies.

### 2.10 Immunohistochemistry

Tissues were fixed in 4% paraformaldehyde, embedded in paraffin, and sectioned. Sections were deparaffinized, rehydrated, and subjected to antigen retrieval. After cooling and blocking with 3% BSA, sections were incubated with primary antibodies overnight at 4 °C. The following day, sections were incubated with an enzyme-conjugated secondary antibody, and signals were developed using a 3,3’-Diaminobenzidine (DAB) substrate. Nuclei were counterstained with hematoxylin. Sections were dehydrated, cleared, and mounted. Images were acquired using a Nikon light microscope, and the percentage of positive cells was quantified with ImageJ software.

### 2.11 Statistical analysis

For comparisons between two independent groups, Student’s *t*-test was used. For longitudinal data involving multiple groups, two-way ANOVA with repeated measures followed by Tukey’s multiple comparisons test was employed. The level of statistical significance is indicated by asterisks (*p< 0.05; **p< 0.01; ***p< 0.001; ns, non-significant).

## 3 Results

### 3.1 Design and modification of AAV capsid targeting DCs

To target cDC1, the peptide CBP-12, which exhibits high affinity for Clec9a, was selected as the ligand. We chose the most widely used AAV2 serotype as the vector, given its extensive clinical data on efficacy and safety. To better expose CBP-12 on the AAV capsid surface, CBP-12 was inserted into the GH loop of AAV VP1 at position R588 the commonly used insertion site (31), thereby altering the natural tropism of AAV while exposing CBP-12. To optimize the display of CBP-12, amino acid mutations were introduced in the regions flanking the insertion site. Additionally, we performed site-directed mutagenesis to convert threonine and serine at positions 491 and 662 to valine (T491V, S662V), aiming to further enhance the efficiency of the engineered AAV vector in target cells. **Figure 1A** illustrates these different modification strategies. Recombinant AAV (rAAV) was produced using a triple-plasmid co-transfection method and purified via ultracentrifugation. Transmission electron microscopy revealed well-dispersed, uniformly shaped viral particles with clear edges and a diameter of approximately 25 nm. Purified AAVs generated via different modification methods exhibited intact morphological structures (**Figure 1B**). Subsequent immunoblotting detected Purified AAVs using antibody specific for AAV2 capsids. The immunoblot bands of AAV capsid proteins with different modification methods did not show significant differences (**Figure 1C**). Further validation in HEK 293T cells using excess virus to ensure maximal infection confirmed that the prepared rAAVs effectively expressed the delivered enhanced green fluorescent protein (EGFP) gene (**Figure 1D**). These results demonstrate that the modified AAVs maintained viral particle integrity and efficiently delivered genes for expression in HEK 293T cells, making them suitable for subsequent experiments. However, no significant differences were observed among the various modification approaches.

**Figure 1:**
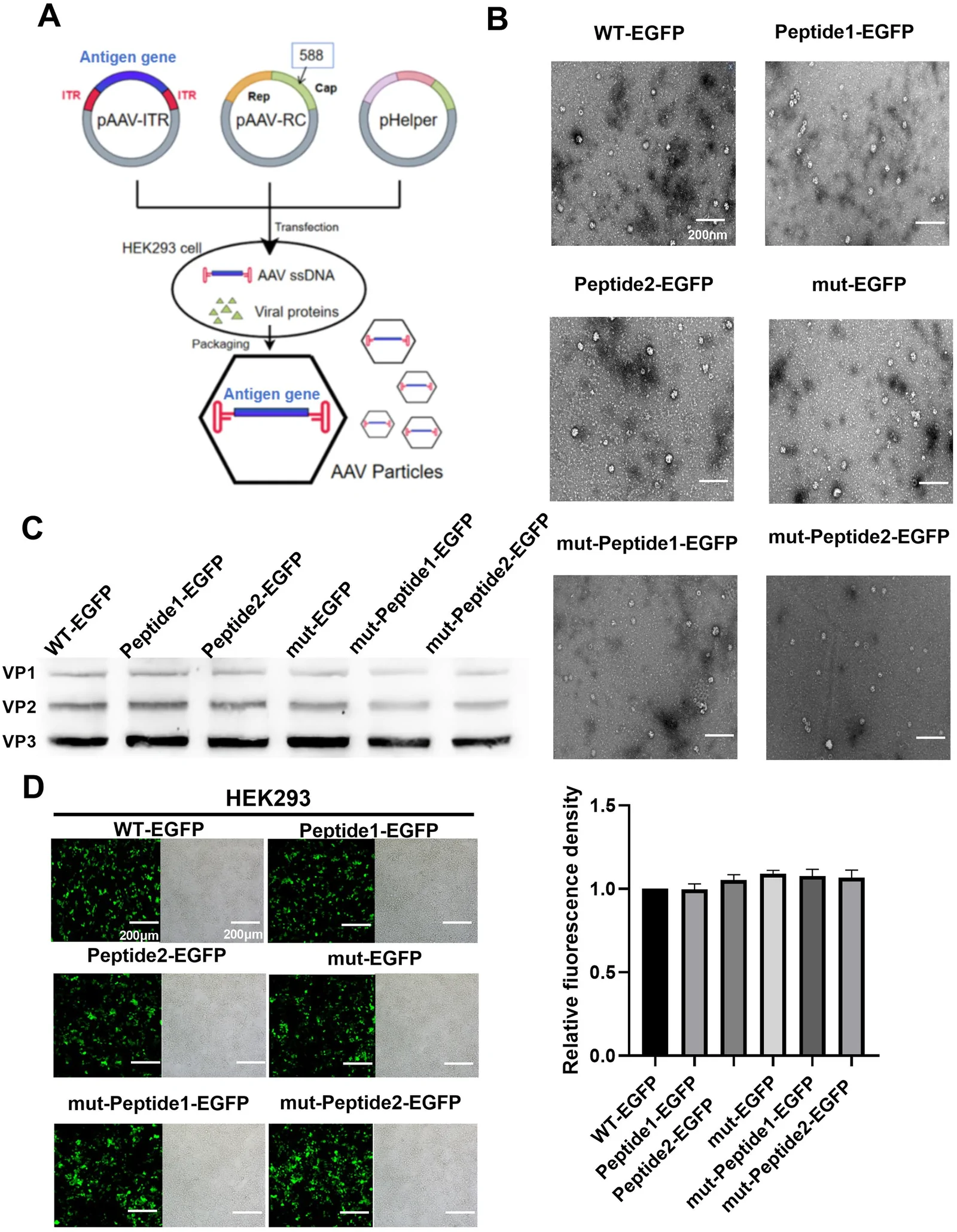
Design and modification of AAV capsid targeting DCs. **(A)** Schematic of different AAV vector modification strategies. **(B)** The morphology of AAV vectors were visualized by the transmission electron microscopy with a negative stain. Images were taken with 30000-fold magnification and the scale bar represents 200 nm. **(C)** Western blot analysis of the indicated AAV vectors. An amount of 10 μL of purified AAV vector stock were heat-denatured in urea buffer before separation with 8% SDS-PAGE and blotting onto a PVDF membrane. AAV viral proteins were detected using the VP1/2/3 antibody (PROGEN). The bar graph shows the densitometric analysis of band intensity. **(D)** EGFP expression analysis at 48 h post-infection at a multiplicity of infection (MOI) of 2000 vgs/cell. Scale bars, 200 µm.

### 3.2 Enhanced gene delivery efficiency of modified rAAV in DC2.4

This study assessed the transduction efficiency of modified rAAV2 variants in DCs through *in vitro* experiments with DC2.4 cells. Since the DC2.4 cells in our laboratory inherently express EGFP, the AAV-delivered EGFP gene was replaced with the OVA gene for these experiments. After infecting DC2.4 cells with different AAV variants at the same titer, the proportion of OVA-positive cells was analyzed using flow cytometry. The results showed that compared to AAV2-wt-OVA, the insertion of CBP-12 significantly enhanced OVA expression in CD11c-positive cells. Furthermore, optimizing the flanking amino acids at the insertion site or introducing T491V and S662V mutations further improved OVA expression efficiency. When these modifications were combined, the proportion of OVA-positive cells in CD11c-positive cells increased by 7.5-fold (**Figure 2A**). RT-qPCR analysis of OVA mRNA levels in DC2.4 cells revealed that the modified AAV variants markedly elevated OVA mRNA abundance, with AAV2-mut-Peptide2-OVA exhibiting the highest abundance (**Figure 2B**). Subsequent Western blot analysis of OVA expression further confirmed that these modifications enhanced OVA expression (**Figure 2C**). In summary, these results indicate that the AAV variants we developed effectively enhance the delivery efficiency of the target gene in DC2.4 cells *in vitro*.

**Figure 2:**
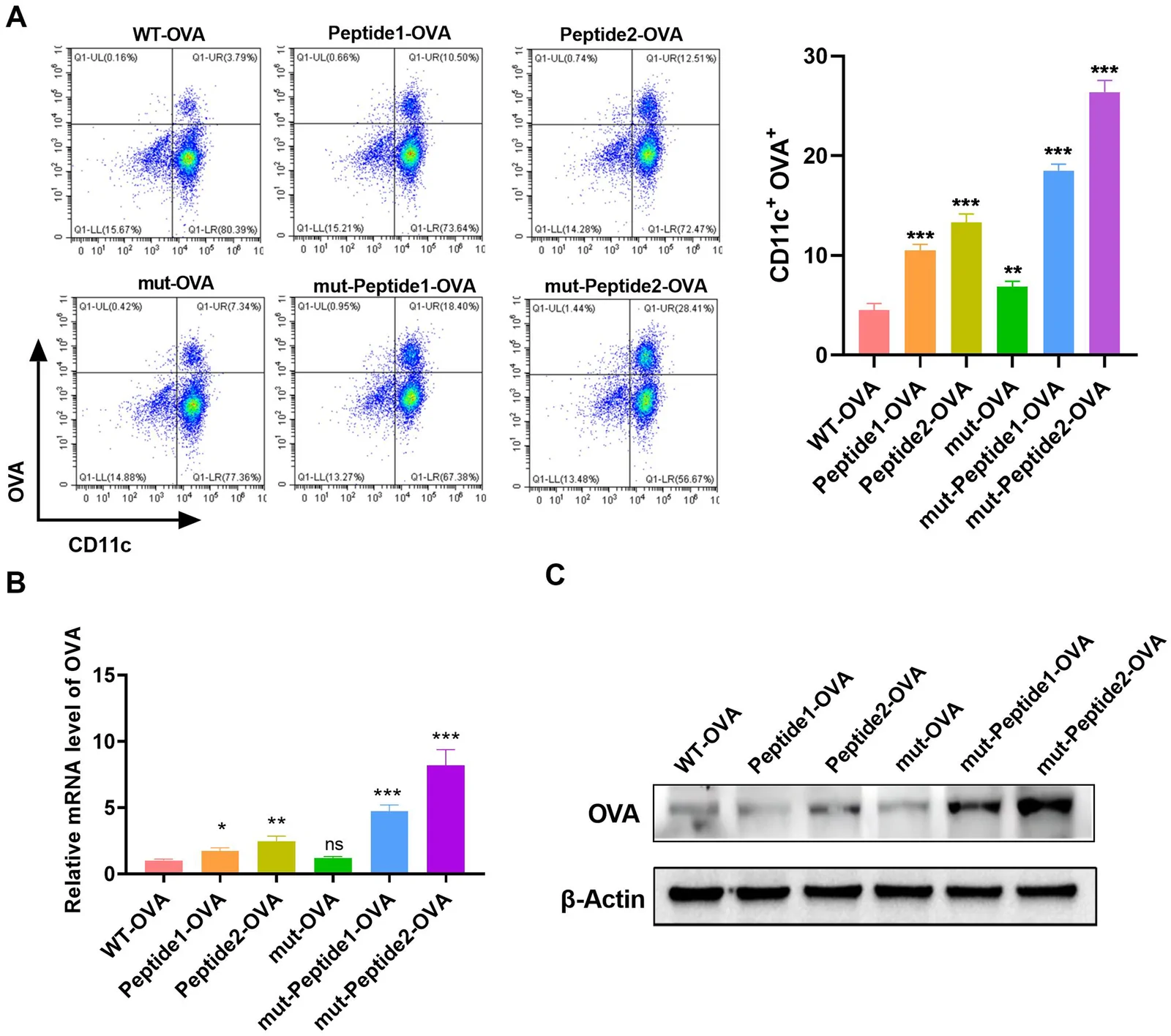
Enhanced gene delivery efficiency of modified rAAV in DC2.4. **(A)** OVA expression on DC2.4 cells was detected 24 h post-transduction via flow cytometry. The bar graph on the right shows the percentage of CD11c^+^OVA^+^ cells. **(B)** DC2.4 cells were collected for OVA mRNA detection by RT-qPCR post-transduction with rAAVs for 24 h. **(C)** Expression of OVA by immunoblotting analysis. DC2.4 cells were infected with rAAV for 48 h. the cells were harvested and lysed. OVA were detected by IB. All experiments were repeated at least three times and similar results were obtained. Differences between groups were assessed using Student *t* test, and statistical significance was denoted by *p < 0.05; **p < 0.01; ***p < 0.001; ns, not significant.

### 3.3 Enhanced gene delivery efficiency of modified rAAV targeting DCs in mice

To further validate the effectiveness of AAV variants for gene delivery to DCs *in vivo*, we administered different AAV variants at the same titer to mice via micro-vein injection and performed analysis three weeks later. We found that compared to AAV-WT-EGFP, the AAV2-mut-Peptide2-EGFP group showed enhanced AAV enrichment and EGFP expression in the lymph nodes. As shown in **Figure 3A**, RT-qPCR and immunoblot analysis of lymph nodes both showed increased levels of AAV-delivered EGFP. Most AAV serotypes are likely to be detected in the liver following intravenous administration, raising concerns about potential hepatotoxicity (28, 32). Correspondingly, we assessed hepatic EGFP levels as an indicator of liver tropism. The modified AAV vectors exhibited only minimal EGFP expression compared to the wild-type controls (**Figure 3B**). This indicates that the engineered AAV, while enhancing transduction efficiency in lymph nodes, presents a further reduced potential for hepatotoxicity compared to the WT vector. Flow cytometric analysis of CD11c-positive cells from selected mouse lymph nodes for the proportion of XCR1 and EGFP positives indicated that the AAV2-mut-Peptide2-EGFP group had the highest percentage of CD11c^+^, XCR1^+^, EGFP^+^ cells relative to the wild-type control and other single modification strategies (**Figure 3C**). Additionally, immunofluorescence examination of mouse lymph node tissues revealed that after administration of AAV2-mut-Peptide2-EGFP, most EGFP signals co-localized with CD11c signals within cells. In other groups, only a fraction of CD11c-positive cells contained EGFP, whereas hardly any significant EGFP signal was observed after infection with the unmodified AAV (**Figure 3D**).

**Figure 3:**
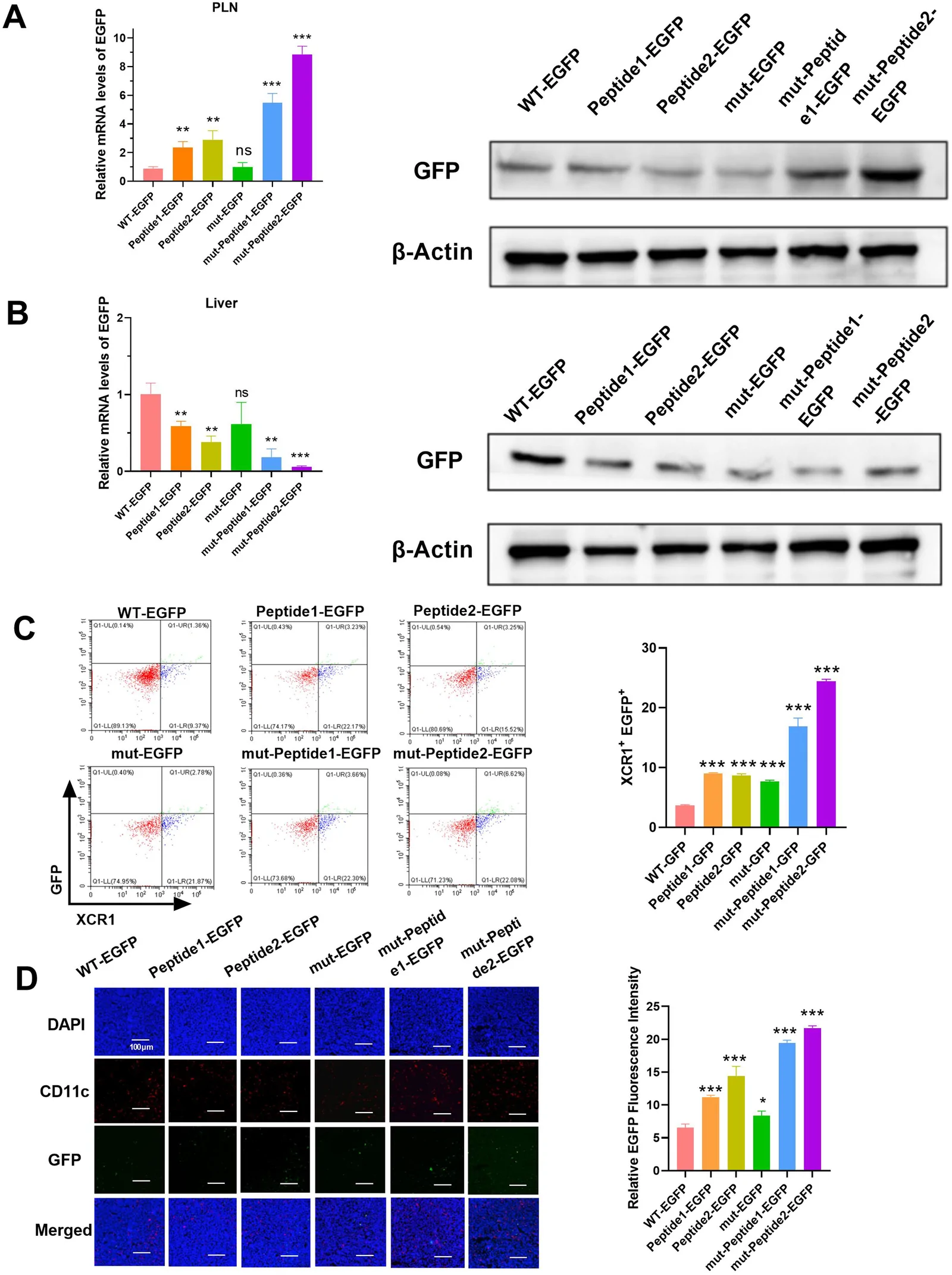
Enhanced gene delivery efficiency of modified rAAV targeting DCs in mice. *In vivo* distribution of rAAV in the popliteal lymph nodes **(A)** and in the liver **(B)** after tail vein injection. EGFP mRNA was detected by RT-qPCR 72 h after injection (*n* = 3 mice, biologically independent samples) and EGFP expression was analyzed using immunoblotting. **(C)** Flow cytometry analysis of the percentage of EGFP^+^ in mouse spleen DCs. **(D)** Immunofluorescence analysis of EGFP expression delivered by rAAV vectors in DCs. Scale bars, 100 µm. Differences between groups were assessed using Student *t* test, and statistical significance was denoted by *p < 0.05; **p < 0.01; ***p < 0.001; ns, not significant.

We further examined EGFP mRNA levels in the heart, lungs, and kidneys by RT-qPCR. No off-target accumulation was observed in these organs. Instead, EGFP mRNA levels were reduced in the lungs and kidneys compared to AAV2-wt-EGFP (**Supplementary Figure S1**). Of note, while minimal EGFP mRNA was detectable in these tissues, reflecting the basal infection level of AAV vectors, these levels were far lower than those detected for AAV2-wt in the liver and for AAV2-mut-Peptide2-EGFP in the lymph nodes. The biosafety of the various AAV variants *in vivo* was further evaluated by monitoring daily changes in mouse body weight. The results showed no significant difference in the body weight growth curves between the AAV-treated groups and the PBS control group (**Supplementary Figure S2**). Furthermore, histological examination revealed no apparent pathological damage, such as significant inflammatory immune cell infiltration, cell necrosis, or tissue structural abnormalities (**Supplementary Figure S3**).

In conclusion, the data demonstrate that our AAV variant can effectively transduce DCs *in vivo* and mediate efficient antigen gene delivery.

### 3.4 OVA as antigen delivered by modified rAAV elicited anti-tumor immune responses in mice

To investigate the efficacy of rAAV vectors in tumor therapy, a subcutaneous hepatocellular carcinoma model was developed in mice using Hepa 1–6 cells stably expressing OVA. The antitumor efficacy was assessed by tail vein injection of rAAV vectors carrying the OVA gene (**Figure 4A**). The tumor volume in the AAV2-mut-Peptide2-OVA group was 254.35 ± 42.88 mm³ on day 12 post-injection, representing a 65% reduction compared to the PBS group and a 44% reduction compared to the AAV2-wt-OVA group carrying the unmodified OVA gene capsid (**Figures 4B–E**). Survival analysis further confirmed that AAV2-mut-Peptide2-OVA significantly prolonged survival compared to PBS controls (**Figure 4D**). These findings demonstrate that AAV2-mut-Peptide2-OVA significantly inhibits hepatocellular carcinoma progression through antigen gene delivery with its effects enhanced by capsid modification. Further assessment of antitumor efficacy was performed via immunohistochemical analysis using Ki67 (**Figure 4F**). As shown, numerous proliferating tumor cells with brown staining were observed in the PBS and AAV2-WT control groups. Notably, the AAV2-mut-Peptide2-OVA group exhibited significant tumor tissue necrosis, with only minimal residual tumor cells detected.

**Figure 4:**
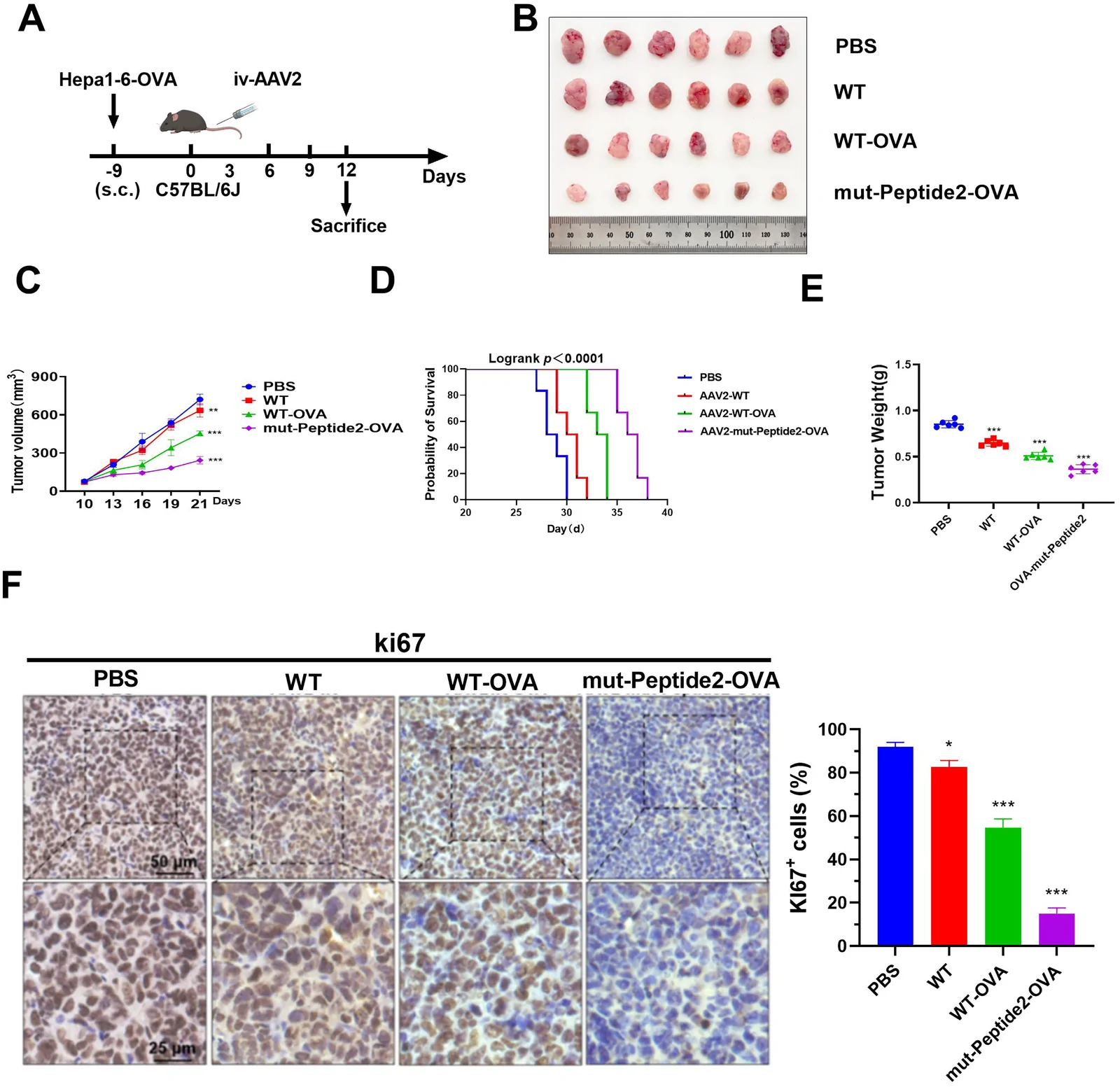
Anti-tumor effect of rAAV vector delivering OVA in Hepa 1-6-OVA mouse model. Hepa 1-6-OVA cells were s.c injected on the right flank of C57BL/6 mice, when tumor volume reached to 80 mm3, mice were randomly grouped (n = 6), and rAAVs were s.c injected in mouse. **(A)** The scheme of tumor model. **(B)** Photographs of the excised tumors after 12 days of treatment. **(C)** Tumor growth curves with the mean tumor volumes of Hepa 1-6-OVA mice model. **(D)** Kaplan-Meier survival curves of tumor-bearing mice. Survival was monitored daily. Statistical significance was determined by log-rank test. **(E)** Tumor weight was measured after sacrificing on D12. **(F)** Ki67 staining of tumor sections after the indicated treatments. Data presented as means ± SEM (n = 3). Tumor growth curves in **(C)** were analyzed using two-way ANOVA with repeated measures followed by Tukey’s multiple comparisons test. All other comparisons were performed using Student *t* test. *p < 0.05; **p < 0.01; ***p < 0.001.

Flow cytometry analysis of CD8^+^ T cell infiltration in the tumor microenvironment revealed that the AAV2-mut-Peptide2-OVA treatment group had the highest percentage of tumor-infiltrating CD8^+^ T cells, approximately doubling that of other groups (**Figure 5A**). This indicates a strong antitumor immune response induced by AAV2-mut-Peptide2-OVA *in vivo*. Consistent with the flow cytometry findings, immunohistochemical analysis of intratumoral CD8 expression revealed a significant increase in the proportion of CD8^+^ T cells in the AAV2-mut-Peptide2-OVA group (**Figure 5B**). Furthermore, we assessed the expression of activated T cells and key immune factors such as IFN-γ, IL-6 and TNF-α (**Figures 5C, D**). CD8^+^IFN-γ^+^ T cells were significantly upregulated in the tumor tissues of the AAV2-mut-Peptide2-OVA group. Additionally, the mRNA levels of IFN-γ, IL-6 and TNF-α were markedly elevated compared to the other three groups. These results suggest that AAV2-mut-Peptide2-OVA effectively promotes the upregulation of immune activation-related factors within the tumor microenvironment, thereby enhancing the host’s antitumor immune efficacy.

**Figure 5:**
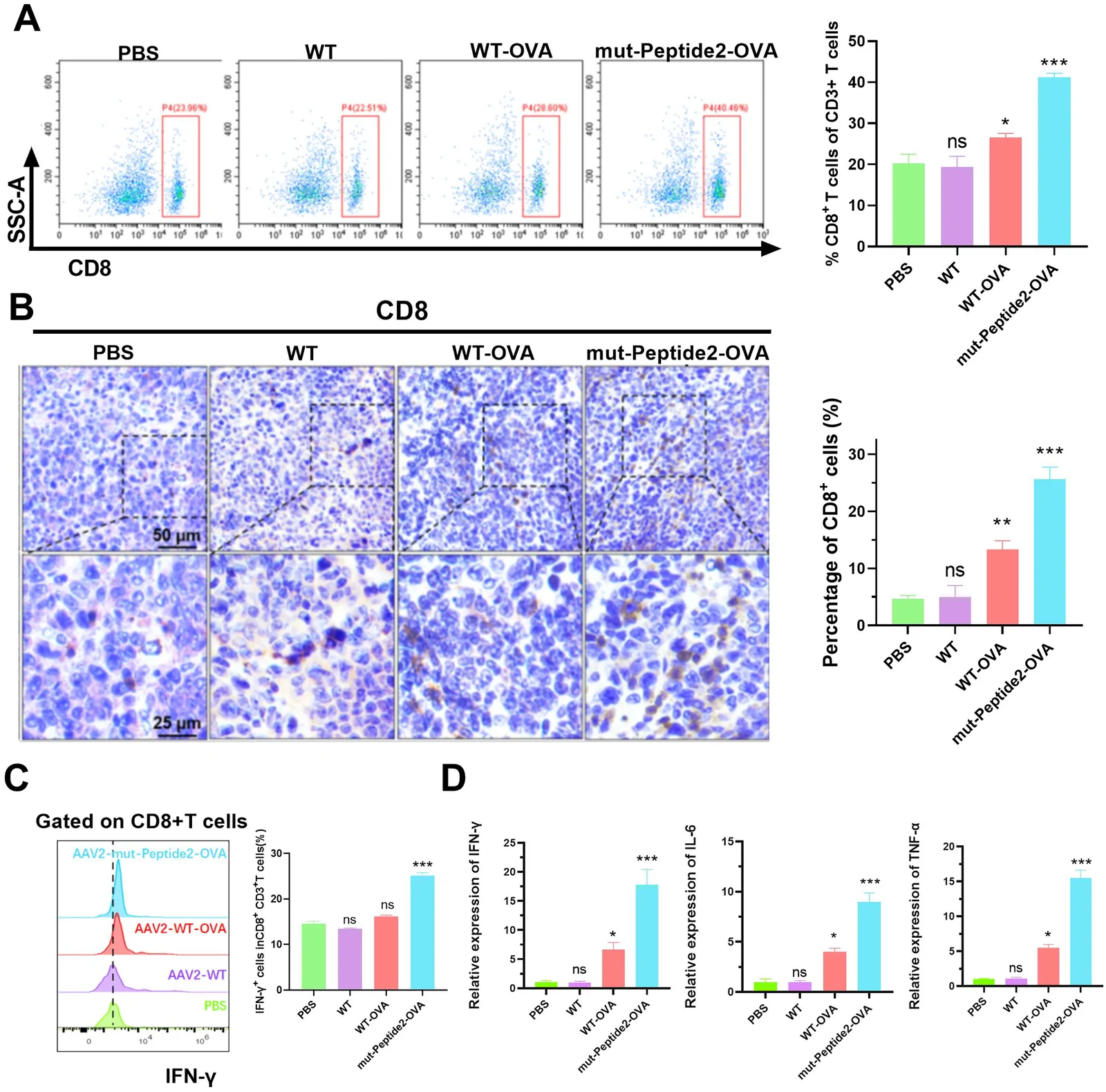
Anti-tumor immune responses in Hepa 1-6-OVA mouse model. **(A)** The proportion of CD8^+^ T cells in tumor tissue were detected by flow cytometry. **(B)** Immunohistochemical analysis of intratumoral CD8 expression. **(C)** The proportion of CD3^+^CD8^+^IFN-γ^+^ T cells in tumor tissue were measured by flow cytometry. **(D)** mRNA expression of pro-inflammatory cytokines IFN-γ, IL-6 and TNF-α in tumor tissue after different treatments. Data were represented as means ± SEM. Statistical significance was determined by unpaired Student *t* test, *p < 0.05; **p < 0.01; ***p < 0.001; ns, not significant.

### 3.5 Modified rAAV delivery of GPC3 inhibits hepatocellular carcinoma subcutaneous tumor progression in mice

Previous studies have demonstrated that Glypican-3 (GPC3) is highly expressed in over 70% of HCC cases but scarcely in healthy liver tissues, positioning it as a promising therapeutic target. GPC3-specific therapies, including bispecific monoclonal antibodies and vaccines, have yielded promising outcomes (33, 34). Our analysis of gene expression profiles in Hepa1–6 cells, compared to normal hepatocyte AML12, confirmed significant GPC3 overexpression as a specific antigen for Hepa1-6. To further explore the efficacy of rAAV vectors in tumor immunotherapy, we utilized a subcutaneous Hepa1–6 mouse model. The GPC3 gene was loaded into a modified rAAV vector as previously described, and the same administration method as in the Hepa1-6-OVA model was employed to assess tumor growth inhibition. In combination with this vector, we also introduced an anti-PD-1 antibody to evaluate the synergistic therapeutic effect. Results indicated that monotherapy with the rAAV vaccine or the anti-PD-1 antibody alone achieved tumor growth inhibition of 63% and 79%, respectively (**Figures 6A–D**). Notably, the combination therapy exhibited significantly more potent efficacy, achieving a 83% reduction in tumor volume by Day 21, which was statistically superior to either monotherapy alone (*p* < 0.001). Survival analysis further demonstrated that combination therapy significantly prolonged survival compared to all monotherapy groups (**Figure 6C**). Immunohistochemical analysis of Ki67 showed reduced proliferation in both monotherapy groups, with the most pronounced reduction observed in the combination group (**Figure 6E**).

**Figure 6:**
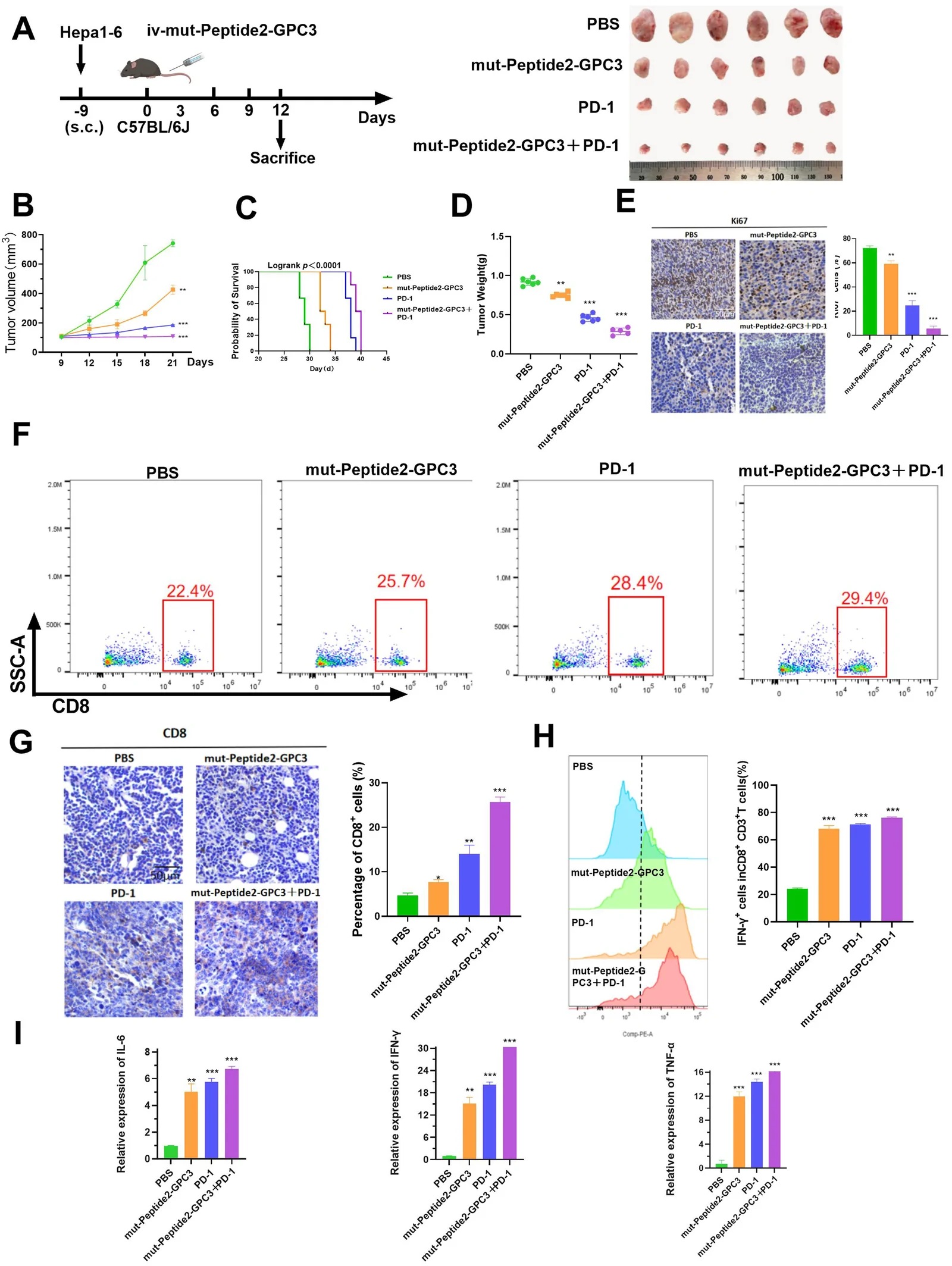
Anti-tumor effect of rAAV vector delivering GPC3 in Hepa 1–6 mouse model. **(A)** Hepa 1–6 cells were s.c injected on the right flank of C57BL/6 mice, mice were randomly grouped (n = 6) after 9 days, and rAAVs were *s.c* injected in mouse. Representative images of tumors after treatment. **(B)** Change of relative tumor volume during the treatment. **(C)** Kaplan-Meier survival curves of tumor-bearing mice. Survival was monitored daily. Statistical significance was determined by log-rank test. **(D)** Tumor weights was measured after sacrificing on D12 after treatment. **(E)** Ki67 staining of tumor sections after the indicated treatments. **(F, G)** The proportion of CD8^+^ T cells in tumor tissue were detected by flow cytometry and immunohistochemistry experiments. **(H)** The proportion of CD3^+^CD8^+^IFN-γ^+^ T cells in tumor tissue. **(I)** mRNA expression of pro-inflammatory cytokines and IL-6, IFN-γ and TNF-α in tumor tissue. Data were represented as means ± SEM. Tumor growth curves in **(B)** were analyzed using two-way ANOVA with repeated measures followed by Tukey’s multiple comparisons test. All other comparisons were performed using Student *t* test. *p < 0.05; **p < 0.01; ***p < 0.001.

Investigation into CD8^+^ T cell infiltration in the tumor microenvironment revealed a significantly higher proportion of CD3^+^CD8^+^ T cells in liver cancer tissues treated with AAV2-mut-Peptide2-GPC3 and/or anti-PD-1 antibody compared to those treated with PBS, as shown by flow cytometry (**Figure 6F**). Immunohistochemical analysis confirmed these findings with consistent intratumoral CD8 expression (**Figure 6G**). We further assessed NK cell infiltration by NKp46 IHC staining. The percentage of NKp46^+^ cells was significantly increased in the AAV2-mut-Peptide2-GPC3 group compared to PBS controls, and combination with anti-PD-1 further enhanced NK cell infiltration (**Supplementary Figure S4**). Additionally, we assessed immune effector molecule expression in the tumor microenvironment, finding significant upregulation of CD8^+^IFN-γ^+^ T cells, IL-6, IFN-γ, and TNF-α in the combination therapy or monotherapy group relative to the PBS group (**Figures 6H, I**). These results indicate that the engineered rAAV targeting DC is an effective vector for tumor immunotherapy, enhancing anti-tumor immune responses.

## 4 Discussion

Targeting DCs has been deemed an attractive strategy to improve the effectiveness of current cancer vaccines technologies (35). This study has developed a novel rAAV-based cancer vaccine platform with a design to target DCs (**Figure 7**). In doing so, the study proposes and validates an AAV vector modification strategy. We selected the widely used and well-characterized AAV2 serotype as the backbone and engineered it by incorporating the high-affinity Clec9a ligand CBP-12 peptide. Further optimization involved refining flanking sequences and introducing key amino acid mutations (T491V/S662V), resulting in a series of rAAV vectors targeting cDC1. Experimental data revealed that the modified AAV variants, particularly AAV2-mut-Peptide2, markedly enhanced antigen gene delivery in DC2.4 cells *in vitro* and efficiently transduced lymph node cDC1 *in vivo*. Additionally, the variants elicited strong antigen-specific anti-tumor immune responses and effectively suppressed tumor growth in a hepatocellular carcinoma model.

**Figure 7:**
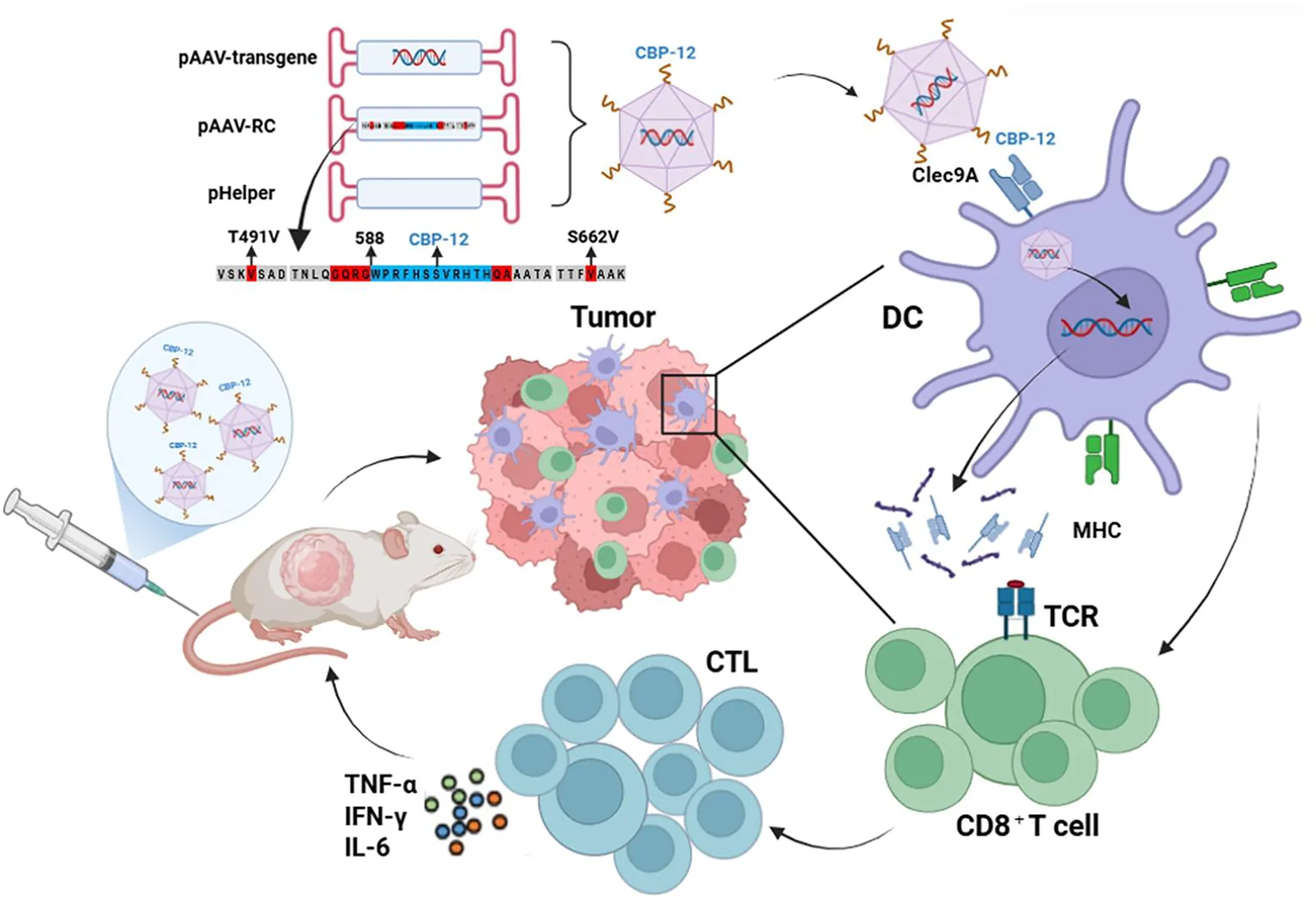
A model depicts that DC-targeting rAAV functions as potent cancer vaccine carriers. This schematic illustrates the engineered rAAV vectors that are designed to target DC for antigen delivery, thereby establishing an antitumor immune response.

This study illustrates the potential of rational AAV capsid engineering to enhance DC-targeting capabilities. The engineered vector demonstrated markedly improved enrichment in murine lymph node cDC1s and increased gene delivery efficiency compared to wild-type AAV2, resulting in stronger anti-tumor T cell responses and improved tumor control. Remarkably, the modified vector outperformed even with the highly immunogenic model antigen OVA. Additionally, when tested with the liver cancer-specific antigen GPC3, the platform successfully induced protective immunity, showcasing its exceptional antigen adaptability and broad application potential.

Compared to ACT such as CAR-T, the vaccine platform proposed in this project eliminates the need for individualized cell preparation, avoiding complex *in vitro* manipulations (6, 36). Moreover, through endogenous antigen expression and cross-presentation, it can induce broader and more durable anti-tumor T-cell responses while potentially significantly reducing the risk of severe adverse effects such as cytokine storms. Notably, the AAV-based viral vector vaccine platform targeting DCs holds tremendous potential for personalized therapy. By integrating tumor neoantigen screening and identification technologies, patient-specific neoantigen gene sequences can be rapidly loaded into targeted AAV vectors, enabling direct and efficient transduction of cDC1 *in vivo* to initiate personalized immune responses against the patient’s unique tumor mutation profile. This strategy bypasses the need for isolating patient cells for complex *in vitro* processing, greatly simplifying the production workflow, thereby offering a novel approach for developing truly “off-the-shelf” personalized tumor vaccines.

Although the AAV2 platform exhibited promising performance in this study, its natural transduction efficiency for DCs is still limited. Exploring AAV serotypes with higher natural affinity for APCs (such as AAV6, AAV8, or AAVrh32.33) as the basis for modification represents a highly promising direction. For instance, AAV6 has been reported to exhibit superior transduction capability for DCs independent of its HSPG-binding properties (37, 38). Using it as a backbone for similar cDC1-targeted modifications may achieve equivalent or even better immune priming effects at lower viral doses.

In terms of targeting strategy, this study validated the efficacy of the CBP-12 peptide targeting Clec9a. However, other specific molecules (e.g., XCR1, CD141) also exist on the surface of DCs and serve as markers of cDC1 (11). Future efforts could focus on identifying novel ligands with higher affinity for Clec9a or developing bispecific/multivalent targeting systems capable of simultaneously engaging multiple cDC1-specific receptors may further optimize the targeting precision and efficiency of the delivery system, thereby more fully unleashing the immunostimulatory potential of cDC1.

A crucial concern in clinical translation is pre-existing immunity. The capsid modification strategy used here inherently aids in partially evading antibody recognition from homologous serotypes. Future research should rigorously assess the efficacy of this modified vector in animal models with pre-existing AAV2 immunity and actively pursue the development of novel capsids with low seroprevalence or enhanced neutralization antibody evasion.

Regarding the durability of the immune response, strong effector T cell responses were observed in the short term in this study. Based on the known property that AAV vectors can mediate long-term stable expression of transgenes, we hypothesized that the immune memory stimulated by this platform may be more durable (26). However, this hypothesis requires formal validation through tumor rechallenge experiments in future studies. We acknowledge that the durability of the anti-tumor immune response and the establishment of immunological memory were not directly assessed in this study. In addition, while sustained expression of antigen is beneficial for maintaining immune memory, there is a potential risk of triggering T-cell depletion, and thus the extent to which effective delivery of AAV-loaded antigen is maintained warrants further exploration.

Given the challenges posed by tumor microenvironment suppression in advanced solid tumors, combining this vaccine platform with other immunotherapies, particularly immune checkpoint inhibitors like anti-PD-1 antibodies, holds significant promise. The observed increase in CD8^+^ T-cell infiltration and IFN-γ upregulation in the tumor microenvironment provides an ideal foundation for synergy with PD-1/PD-L1 inhibitors. This was further confirmed by the results from **Figure 6**. The combination, where the vaccine “initiates” and “expands” tumor-specific T cells and the checkpoint inhibitor “unsuppresses” these T cells at the tumor site, is anticipated to overcome drug resistance and enhance efficacy.

In conclusion, this study developed and validated a novel cDC1-targeted rAAV as potent cancer vaccine carriers, offering a potential solution to the limitations of current cancer vaccines.

## Data Availability

The original contributions presented in the study are included in the article/**Supplementary Material**. Further inquiries can be directed to the corresponding authors.
