# Supplementary material for: Dendritic-cell-targeting adeno-associated virus as potent cancer vaccine carriers
Source: Front Immunol. 2026 Sep 30;17:1932907. doi: 10.3389/fimmu.2026.1932907 (PMC13662115; doi:10.3389/fimmu.2026.1932907)
Supplement: Supplementary file 1 [file Table1.docx]

Supplementary Material

**Supplementary Tables**

**Table S1. Amino acid sequence of various modified AAV VP1 in this study.**

WT AAV VP1

MAADGYLPDWLEDTLSEGIRQWWKLKPGPPPPKPAERHKDDSRGLVLPGYKYLGPFNGLDKGEPVNEADAAAVEHDKAYDRQLDSGDNPYLKYNHADAEFQERLKEDTSFGGNLGRAVFQAKKRVLEPLGLVEEPVKTAPGKKRPVEHSPVEPDSSSGTGKAGQQPARKRLNFGQTGDADSVPDPQPLGQPPAAPSGLGTNTMATGSGAPMADNNEGADGVGNSSGNWHCDSTWMGDRVITTSTRTWALPTYNNHLYKQISSQSGASNDNHYFGYSTPWGYFDFNRFHCHFSPRDWQRLINNNWGFRPKRLNFKLFNIQVKEVTQNDGTTTIANNLTSTVQVFTDSEYQLPYVLGSAHQGCLPPFPADVFMVPQYGYLTLNNGSQAVGRSSFYCLEYFPSQMLRTGNNFTFSYTFEDVPFHSSYAHSQSLDRLMNPLIDQYLYYLSRTNTPSGTTTQSRLQFSQAGASDIRDQSRNWLPGPCYRQQRVSKTSADNNNSEYSWTGATKYHLNGRDSLVNPGPAMASHKDDEEKFFPQSGVLIFGKQGSEKTNVDIEKVMITDEEEIRTTNPVATEQYGSVSTNLQRGNRQAATADVNTQGVLPGMVWQDRDVYLQGPIWAKIPHTDGHFHPSPLMGGFGLKHPPPQILIKNTPVPANPSTTFSAAKFASFITQYSTGQVSVEIEWELQKENSKRWNPEIQYTSNYNKSVNVDFTVDTNGVYSEPRPIGTRYLTRNL

Peptide1 AAV VP1

MAADGYLPDWLEDTLSEGIRQWWKLKPGPPPPKPAERHKDDSRGLVLPGYKYLGPFNGLDKGEPVNEADAAAVEHDKAYDRQLDSGDNPYLKYNHADAEFQERLKEDTSFGGNLGRAVFQAKKRVLEPLGLVEEPVKTAPGKKRPVEHSPVEPDSSSGTGKAGQQPARKRLNFGQTGDADSVPDPQPLGQPPAAPSGLGTNTMATGSGAPMADNNEGADGVGNSSGNWHCDSTWMGDRVITTSTRTWALPTYNNHLYKQISSQSGASNDNHYFGYSTPWGYFDFNRFHCHFSPRDWQRLINNNWGFRPKRLNFKLFNIQVKEVTQNDGTTTIANNLTSTVQVFTDSEYQLPYVLGSAHQGCLPPFPADVFMVPQYGYLTLNNGSQAVGRSSFYCLEYFPSQMLRTGNNFTFSYTFEDVPFHSSYAHSQSLDRLMNPLIDQYLYYLSRTNTPSGTTTQSRLQFSQAGASDIRDQSRNWLPGPCYRQQRVSKTSADNNNSEYSWTGATKYHLNGRDSLVNPGPAMASHKDDEEKFFPQSGVLIFGKQGSEKTNVDIEKVMITDEEEIRTTNPVATEQYGSVSTNLQRGNRWPRFHSSVRHTHQAATADVNTQGVLPGMVWQDRDVYLQGPIWAKIPHTDGHFHPSPLMGGFGLKHPPPQILIKNTPVPANPSTTFSAAKFASFITQYSTGQVSVEIEWELQKENSKRWNPEIQYTSNYNKSVNVDFTVDTNGVYSEPRPIGTRYLTRNL

Peptide2 AAV VP1

MAADGYLPDWLEDTLSEGIRQWWKLKPGPPPPKPAERHKDDSRGLVLPGYKYLGPFNGLDKGEPVNEADAAAVEHDKAYDRQLDSGDNPYLKYNHADAEFQERLKEDTSFGGNLGRAVFQAKKRVLEPLGLVEEPVKTAPGKKRPVEHSPVEPDSSSGTGKAGQQPARKRLNFGQTGDADSVPDPQPLGQPPAAPSGLGTNTMATGSGAPMADNNEGADGVGNSSGNWHCDSTWMGDRVITTSTRTWALPTYNNHLYKQISSQSGASNDNHYFGYSTPWGYFDFNRFHCHFSPRDWQRLINNNWGFRPKRLNFKLFNIQVKEVTQNDGTTTIANNLTSTVQVFTDSEYQLPYVLGSAHQGCLPPFPADVFMVPQYGYLTLNNGSQAVGRSSFYCLEYFPSQMLRTGNNFTFSYTFEDVPFHSSYAHSQSLDRLMNPLIDQYLYYLSRTNTPSGTTTQSRLQFSQAGASDIRDQSRNWLPGPCYRQQRVSKTSADNNNSEYSWTGATKYHLNGRDSLVNPGPAMASHKDDEEKFFPQSGVLIFGKQGSEKTNVDIEKVMITDEEEIRTTNPVATEQYGSVSTNLQGQRGWPRFHSSVRHTHQAAATADVNTQGVLPGMVWQDRDVYLQGPIWAKIPHTDGHFHPSPLMGGFGLKHPPPQILIKNTPVPANPSTTFSAAKFASFITQYSTGQVSVEIEWELQKENSKRWNPEIQYTSNYNKSVNVDFTVDTNGVYSEPRPIGTRYLTRNL

mut AAV VP1

MAADGYLPDWLEDTLSEGIRQWWKLKPGPPPPKPAERHKDDSRGLVLPGYKYLGPFNGLDKGEPVNEADAAAVEHDKAYDRQLDSGDNPYLKYNHADAEFQERLKEDTSFGGNLGRAVFQAKKRVLEPLGLVEEPVKTAPGKKRPVEHSPVEPDSSSGTGKAGQQPARKRLNFGQTGDADSVPDPQPLGQPPAAPSGLGTNTMATGSGAPMADNNEGADGVGNSSGNWHCDSTWMGDRVITTSTRTWALPTYNNHLYKQISSQSGASNDNHYFGYSTPWGYFDFNRFHCHFSPRDWQRLINNNWGFRPKRLNFKLFNIQVKEVTQNDGTTTIANNLTSTVQVFTDSEYQLPYVLGSAHQGCLPPFPADVFMVPQYGYLTLNNGSQAVGRSSFYCLEYFPSQMLRTGNNFTFSYTFEDVPFHSSYAHSQSLDRLMNPLIDQYLYYLSRTNTPSGTTTQSRLQFSQAGASDIRDQSRNWLPGPCYRQQRVSKVSADNNNSEYSWTGATKYHLNGRDSLVNPGPAMASHKDDEEKFFPQSGVLIFGKQGSEKTNVDIEKVMITDEEEIRTTNPVATEQYGSVSTNLQRGNRQAATADVNTQGVLPGMVWQDRDVYLQGPIWAKIPHTDGHFHPSPLMGGFGLKHPPPQILIKNTPVPANPSTTFVAAKFASFITQYSTGQVSVEIEWELQKENSKRWNPEIQYTSNYNKSVNVDFTVDTNGVYSEPRPIGTRYLTRNL

mut-Peptide1 AAV VP1

MAADGYLPDWLEDTLSEGIRQWWKLKPGPPPPKPAERHKDDSRGLVLPGYKYLGPFNGLDKGEPVNEADAAAVEHDKAYDRQLDSGDNPYLKYNHADAEFQERLKEDTSFGGNLGRAVFQAKKRVLEPLGLVEEPVKTAPGKKRPVEHSPVEPDSSSGTGKAGQQPARKRLNFGQTGDADSVPDPQPLGQPPAAPSGLGTNTMATGSGAPMADNNEGADGVGNSSGNWHCDSTWMGDRVITTSTRTWALPTYNNHLYKQISSQSGASNDNHYFGYSTPWGYFDFNRFHCHFSPRDWQRLINNNWGFRPKRLNFKLFNIQVKEVTQNDGTTTIANNLTSTVQVFTDSEYQLPYVLGSAHQGCLPPFPADVFMVPQYGYLTLNNGSQAVGRSSFYCLEYFPSQMLRTGNNFTFSYTFEDVPFHSSYAHSQSLDRLMNPLIDQYLYYLSRTNTPSGTTTQSRLQFSQAGASDIRDQSRNWLPGPCYRQQRVSKVSADNNNSEYSWTGATKYHLNGRDSLVNPGPAMASHKDDEEKFFPQSGVLIFGKQGSEKTNVDIEKVMITDEEEIRTTNPVATEQYGSVSTNLQRGNRWPRFHSSVRHTHQAATADVNTQGVLPGMVWQDRDVYLQGPIWAKIPHTDGHFHPSPLMGGFGLKHPPPQILIKNTPVPANPSTTFVAAKFASFITQYSTGQVSVEIEWELQKENSKRWNPEIQYTSNYNKSVNVDFTVDTNGVYSEPRPIGTRYLTRNL

mut-Peptide2 AAV VP1

MAADGYLPDWLEDTLSEGIRQWWKLKPGPPPPKPAERHKDDSRGLVLPGYKYLGPFNGLDKGEPVNEADAAAVEHDKAYDRQLDSGDNPYLKYNHADAEFQERLKEDTSFGGNLGRAVFQAKKRVLEPLGLVEEPVKTAPGKKRPVEHSPVEPDSSSGTGKAGQQPARKRLNFGQTGDADSVPDPQPLGQPPAAPSGLGTNTMATGSGAPMADNNEGADGVGNSSGNWHCDSTWMGDRVITTSTRTWALPTYNNHLYKQISSQSGASNDNHYFGYSTPWGYFDFNRFHCHFSPRDWQRLINNNWGFRPKRLNFKLFNIQVKEVTQNDGTTTIANNLTSTVQVFTDSEYQLPYVLGSAHQGCLPPFPADVFMVPQYGYLTLNNGSQAVGRSSFYCLEYFPSQMLRTGNNFTFSYTFEDVPFHSSYAHSQSLDRLMNPLIDQYLYYLSRTNTPSGTTTQSRLQFSQAGASDIRDQSRNWLPGPCYRQQRVSKVSADNNNSEYSWTGATKYHLNGRDSLVNPGPAMASHKDDEEKFFPQSGVLIFGKQGSEKTNVDIEKVMITDEEEIRTTNPVATEQYGSVSTNLQGQRGWPRFHSSVRHTHQAAATADVNTQGVLPGMVWQDRDVYLQGPIWAKIPHTDGHFHPSPLMGGFGLKHPPPQILIKNTPVPANPSTTFVAAKFASFITQYSTGQVSVEIEWELQKENSKRWNPEIQYTSNYNKSVNVDFTVDTNGVYSEPRPIGTRYLTRNL

**Table S2. Primers used for RT-qPCR assay.**

| **Gene** | **Forward sequence** | **Reverse sequence** |
| --- | --- | --- |
| *EGFP* | GACCACATGAAGCAGCACGACTTCTTCAAG | GTTGTGGCTGTTGTAGTTGTACTCCAGCT |
| *OVA* | ATGCTGAAGAGAGATACCCAATCCTGCCA | GTCACTCTGAAAGGCATTGCTTGTGTGTC |
| *TNF-α* | ACTGGCAGAAGAGGCACTCC | GCCACAAGCAGGAATGAGAA |
| *IFN-γ* | CTGGAGGAACTGGCAAAAGGATGG | GACGCTTATGTTGTTGCTGATGGC |
| *IL-6* | CTTCTTGGGACTGATGCTGGTGAC | TCTGTTGGGAGTGGTATCCTCTGTG |

**Supplementary Figures**

**Figure S1. RT-qPCR analysis of AAV distribution in non-target organs.** EGFP mRNA levels in the heart (A), lungs (B), and kidneys (C) were quantified by RT-qPCR 72 h after tail vein injection. Data are presented as means ± SEM (n = 3). Statistical significance was determined by unpaired Student *t* test, *, *p*<0.05; ns, not significant.

**Figure S2. Mice body weight changes.** Systemic evaluation of the in vivo biosafety of the rAAVs. Mice received rAAVs at the therapeutic dose and via the injection route described in the main text, and body weight was monitored every two days to assess overall health status.

**Figure S3. HE staining of major organs from mice injected with different rAAV vectors.** Mice received rAAV at the therapeutic dose and via the injection route described in the main text. Three weeks later, major organs (liver, spleen, lung, kidney, and heart) were collected and subjected to HE staining to evaluate biosafety.

**Figure S4. NKp46 IHC staining of tumor tissues from the Hepa1-6 model.** Representative images and quantification of NKp46^+^ cells in PBS, AAV2-mut-Peptide2-GPC3, anti-PD-1, and combination therapy groups. Scale bars 50 μm. Data were represented as means ± SEM. Statistical significance was determined by unpaired Student *t* test, ***, *p*<0.001.

**Figure S5. Gating strategy for flow cytometry experiments.** Representative gating plots are shown for: (A) Flow cytometry analysis of OVA expression on DC2.4 cells (Figure 2A). (B) Flow cytometry analysis of the percentage of EGFP^+^ cells in mouse spleen DCs (Figure 3C). (C) CD8^+^ tumor-infiltrating T cells from the Hepa 1-6-OVA model (Figure 5A). (D) Flow cytometry analysis of the proportion of CD3^+^CD8^+^IFN-γ^+^ T cells in tumor tissue (Figure 5C). (E) CD8^+^ tumor-infiltrating T cells from Hepa 1-6 model (Figure 6E). (F) The proportion of CD3^+^CD8^+^IFN-γ^+^ T cells in tumor tissue (Figure 6G). The same gating strategy and parameter settings were applied to all treatment groups.
